# Analgesic tone conferred by constitutively active mu opioid receptors in mice lacking β-arrestin 2

**DOI:** 10.1186/1744-8069-7-24

**Published:** 2011-04-12

**Authors:** Hoa Lam, Matthew Maga, Amynah Pradhan, Christopher J Evans, Nigel T Maidment, Tim G Hales, Wendy Walwyn

**Affiliations:** 1 Department of Psychiatry and Biobehavioral Sciences, Stefan Hatos Center for Neuropharmacology, Semel Institute, University of California, Los Angeles, CA 90095, USA; 2 Institute of Academic Anaesthesia, Centre for Neuroscience, University of Dundee, Dundee DD1 9SY, UK

## Abstract

Hedonic reward, dependence and addiction are unwanted effects of opioid analgesics, linked to the phasic cycle of μ opioid receptor activation, tolerance and withdrawal. *In vitro *studies of recombinant G protein coupled receptors (GPCRs) over expressed in cell lines reveal an alternative tonic signaling mechanism that is independent of agonist. Such studies demonstrate that constitutive GPCR signaling can be inhibited by inverse agonists but not by neutral antagonists. However, ligand-independent activity has been difficult to examine *in vivo*, at the systems level, due to relatively low levels of constitutive activity of most GPCRs including μ receptors, often necessitating mutagenesis or pharmacological manipulation to enhance basal signaling. We previously demonstrated that the absence of β-arrestin 2 (β-arr2) augments the constitutive coupling of μ receptors to voltage-activated Ca^2+ ^channels in primary afferent dorsal root ganglion neurons from β-arr2-/- mice. We used this *in vitro *approach to characterize neutral competitive antagonists and inverse agonists of the constitutively active wild type μ receptors in neurons. We administered these agents to β-arr2-/- mice to explore the role of constitutive μ receptor activity in nociception and hedonic tone. This study demonstrates that the induction of constitutive μ receptor activity *in vivo *in β-arr2-/- mice prolongs tail withdrawal from noxious heat, a phenomenon that was reversed by inverse agonists, but not by antagonists that lack negative efficacy. By contrast, the aversive effects of inverse agonists were similar in β-arr2-/- and β-arr2+/+ mice, suggesting that hedonic tone was unaffected.

## Introduction

Costa and Herz first demonstrated agonist independent opioid receptor signaling in the membranes of NG-108-15 neuroblastoma cells by assaying GTPase activity [1]. They identified δ opioid receptor ligands that inhibit the actions of agonists but have minimal inhibitory effects on basal δ receptor activity. These agents are neutral competitive antagonists. By contrast, ligands that inhibit both basal signaling and agonist-evoked signaling are inverse agonists, drugs that exhibit negative intrinsic efficacy.

μ Opioid receptors exhibit low levels of constitutive activity and therefore several studies have employed strategies of over-expression, mutagenesis and/or pharmacological manipulation in order to enhance basal signaling [2-6]. Initial studies examining constitutive activity of the μ receptor measured inverse agonist induced reductions in GTP-γ-S binding or cAMP accumulation in cell lines over-expressing recombinant receptors [5-8]. These studies established that naloxone and naltrexone have negative efficacy. By contrast, the hydroxyl derivatives of both naloxone (6α- and 6β-naloxol) and naltrexone (6β-naltrexol) have minimal negative efficacy and are therefore considered to be neutral antagonists [9,10]. Prolonged morphine treatment *in vivo *increases μ receptor constitutive activity in the striatum of morphine dependent mice [11] and this is associated with enhanced naloxone induced aversion which persists after cessation of morphine administration [12,13]. These studies suggest that an agonist-induced induction of μ receptor constitutive activity disrupts hedonic homeostasis.

In addition to inhibiting adenylyl cyclase, active opioid receptors inhibit high threshold voltage dependent Ca^2+ ^channels (VDCCs) and activate K^+ ^channels [14]. Coupling to all three effectors occurs through inhibitory G-proteins, which upon activation dissociate into component Gαi/o and βγ subunits. The βγ subunits bind to N- and P/Q-type VDCCs directly inhibiting Ca^2+ ^entry in a voltage-dependent fashion [15]. Strong depolarization reverses the interaction of the βγ subunits with VDCCs causing a facilitation of current amplitude that represents reversal of inhibition. Voltage-dependent reversal of basal inhibition of VDCCs by μ receptors provides an assay for constitutive activity in neurons [16]. We demonstrated that DRG neurons from mice lacking β-arrestin 2 (β-arr2) exhibited constitutive μ receptor inhibition of VDCCs, revealed by an enhancement of voltage-dependent facilitation compared to that observed in recordings from β-arr2+/+ neurons [17]. The inverse agonist naltrexone inhibited facilitation while the neutral antagonist CTAP had no effect. The peptide CTAP has limited bioavailability *in vivo *therefore in this study we used β-arr2-/- DRG neurons to establish the relative intrinsic efficacies of the alkaloids naloxone, 6α-naloxol, 6β-naloxol and 6β-naltrexol in peripheral nociceptors. Having characterized these agents *in vitro *we used them to probe a role for constitutively active μ receptors nociception.

In agreement with a previous finding [18] we demonstrated that β-arr2-/- mice exhibit enhanced basal thermal analgesia compared to β-arr2+/+ mice. Our findings suggest that basal thermal analgesia in β-arr2-/- mice is mediated by constitutively active μ receptors. By contrast, there was no difference in hedonic homeostasis between β-arr2-/- and β-arr2+/+ mice assessed using naloxone-evoked conditioned place aversion.

## Methods

### Cell culture

DRGs from all spinal levels were harvested from early postnatal (p0-1) or adult mice (4-6 weeks old), which contained both (β-arr2+/+) or neither (β-arr2-/-) of the β-arr2 alleles in the C57BL/6 background. DRGs were dissociated in trypsin (Invitrogen, Carlsbad, CA) for the early postnatal neurons, or Collagenase (Liberase TH and TM, Roche, Indianapolis, IN) for the adult neurons. 1 × 10^4 ^cells were plated on each poly-D-lysine (Sigma, MO) and laminin (Invitrogen, CA) coated coverslip of MatTek dishes, (Ashland, MA) as previously described [17].

### Electrophysiology

After 24-48 h in vitro, the whole-cell patch-clamp (Axopatch 200A amplifier, Axon Instruments Inc., CA) technique was used to record VDCC activity from small DRG neurons (capacitance less than 15 pF). The external solution contained (in mM): 130 TEA-Cl, 10 CaCl_2_, 5 HEPES, 25 D-glucose and 0.25 tetrodotoxin (pH 7.2). Recording electrodes contained (in mM): 105 CsCl, 40 HEPES, 5 D-glucose, 2.5 MgCl_2_, 10 EGTA, 2 Mg^2+^-ATP and 0.5 Na^+^-GTP (pH 7.2). VDCCs were activated using voltage-steps from -80 mV to 10 mV at 20 s intervals. Such steps were carried out in the absence or presence of a prepulse to 80 mV, a 2 step-protocol. Voltage-activated currents were low-pass filtered at 2 KHz, digitized (Digidata, Axon Instruments Inc., CA) at 10 KHz, and stored on a PC. Leak currents were nulled using the P/4 subtraction method. All ligands were diluted in the external solution on the day of the experiment and applied through the perfusion system at ~10 ml/min. Mean current amplitudes were measured (pCLAMP 9.0, Molecular Devices, CA) between 5 and 10 ms after initiating the depolarizing step. Once a stable recording was obtained, Ca^2+ ^in the external solution was replaced by Ba^2+ ^(10 mM) to minimize Ca^2+ ^current inactivation.

### Behavioral experiments

For behavioral experiments male and female 2-3 month old progeny of heterozygous matings of mice fully backcrossed into the C57Bl/6 background were used to test thermal or mechanical analgesia or place conditioning, each mouse undergoing a single test. Thermal analgesia was also tested in double knockout mice lacking both μ receptors and β-arrestin 2 generated from heterozygous matings of both lines, both of which were fully back-crossed into the C57Bl/6 background. All animal research was conducted in accordance with the federal regulations as set forth in the Animal Welfare Act (AWA), the 1996 *Guide for the Care and Use of Laboratory Animals*, PHS Policy for the Humane Care and Use of Laboratory Animals, as well as UCLA's policies and procedures as set forth in the UCLA Animal Care and Use Training Manual.

### Nociception

Thermal nociception was measured by either the tail immersion [19] or the Hargreaves [20] test and mechanical nociception by von Frey filaments [21]. For all tests animals were habituated to the test environment for 2 days prior to testing which took place between 8.00 and 16.00 h. For the tail immersion test the last 2.5 cm of the tail was immersed in 48°C water and the latency for tail withdrawal measured. The maximum immersion time was 15 s. After 3 basal measurements, saline or the test drug was injected subcutaneously (sc) at a volume of 10 μl/g body weight (bw) and the time to respond measured 30, 60 and 90 min thereafter. The compounds tested and doses used were (in mg/kg): naloxone (0.5), 6α-naloxol (1.0), 6β-naloxol (10), naltrexone (0.5) and 6β-naltrexol (10). Naloxone and naltrexone were obtained from Sigma (MO) and the hydroxyl derivatives from the National Institute on Drug Abuse. For the Hargreaves test each mouse was habituated to the 10 cm × 10 cm × 15 cm enclosure on a heated (22°C) glass plate for 20 min prior to testing. A medium intensity infrared beam was shone on the rear foot pad of the hind paw and the latency to lift the paw was measured. The average responses of both left and right hind paws were obtained for each data-point with a minimum interval of 5 min between testing either paw. After the baseline measurements were obtained naloxone was injected (0.5 mg/kg) and the response measured 30 min later. For mechanical nociception the response threshold to punctate mechanical stimuli was tested according to the up-and-down method [21] as previously described [22]. In this case, the plantar surface of one hind paw was stimulated with a series of eight von Frey filaments (bending force ranging from 0.01 to 2 g). A response was defined as a lifting or shaking of the paw upon stimulation.

### Place conditioning

The conditioning apparatus (Coulbourn Instruments, Allentown, PA, USA) and experimental protocol have been described previously [23]. Briefly, arenas were divided into three distinctively patterned chambers: a neutral start chamber and two conditioning chambers, which were discernable by pattern and odor. The drug-paired chamber was randomized across subjects and treatments. On day 1 (habituation session), mice were placed in the start chamber, permitted access to the entire apparatus for 15 minutes, and time spent in each chamber was recorded. On the mornings of days 2-4 (conditioning sessions), animals received saline (10 μl/g bw sc) and were confined to the 'vehicle-paired' chamber for 30 minutes. Four hours later, the same mice received a single naloxone injection (0.25, 0.5, 1.0, or 10 mg/kg sc) or vehicle and were confined to the 'drug-paired' chamber for 30 min. On day 5 (test session), animals, in a drug-free state, were placed in the neutral chamber and permitted to explore the entire apparatus for 15 minutes, with time spent in each chamber recorded. Separate groups of β-arr2-/- and β-arr2+/+ mice were used for each dose of naloxone.

### Statistical analysis

Nociception assays involved sample sizes of 9-22 mice per group. Data from these and the electrophysiological assays were analyzed by one way ANOVA for genotype, and the *post hoc *Scheffe test. Data are presented as the mean ± SEM of the time to respond. For place conditioning analysis of initial bias between future drug-paired versus future vehicle-paired chamber was conducted by three way ANOVA with factors of genotype, dose, and the repeated-measures factor of chamber. Statistical comparisons of times spent in the drug-paired chamber between habituation and test day were made using three way ANOVA with factors of genotype, dose, and the repeated-measures factor of day (habituation versus test).

## Results

### Constitutive μ receptor coupling to VDCCs in small β-arr2-/- neurons from postnatal and adult mice

**Figure 1:**
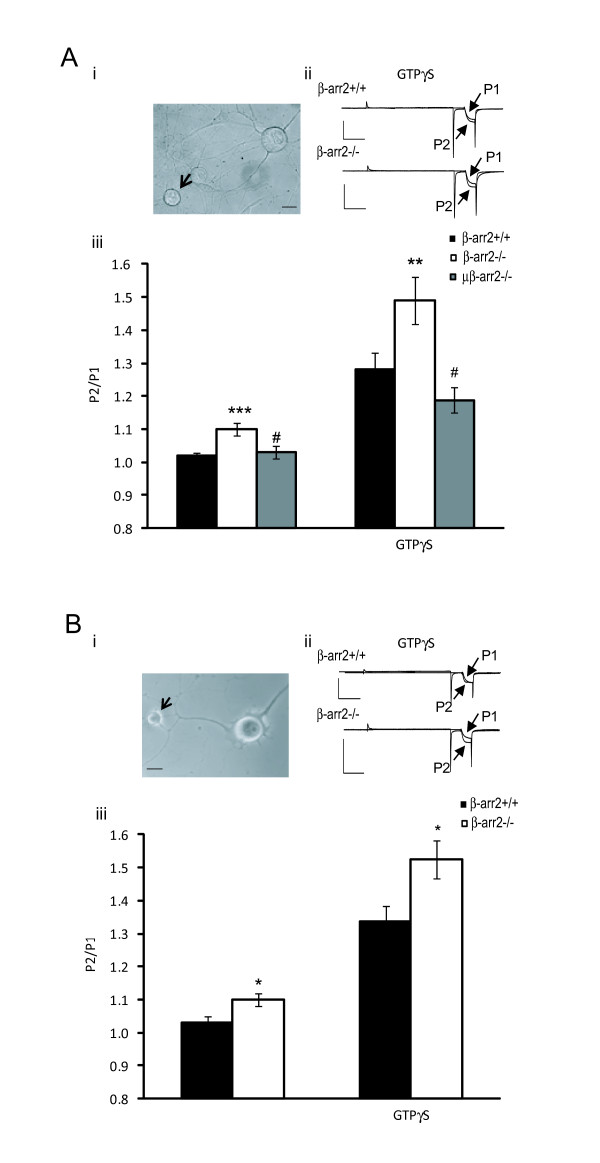
**Increased facilitation in early post-natal and adult DRG neurons**. An increase in Ba^2+ ^current facilitation following a depolarizing pre-pulse to 80 mV is consistent with constitutive coupling of GPCRs with voltage-dependent Ca^2+ ^channels (VDCCs) through Gβγ subunits. Facilitation was quantified by a two-step protocol comparing the current amplitude with (P2) or without a pre-pulse (P1), the P2/P1 ratio. A. Early post-natal DRG neurons i. Whole cell patch clamp recordings for all experiments were conducted in small DRGs (highlighted by the arrow, the scale-bar represents 10 μM), the population of cells most likely to express the μ receptor. ii. Exemplar recordings from β-arr2+/+ and β-arr2-/- cells, in which GTP-γ-S was included in the intracellular recording solution, demonstrate the relative increase in current following a depolarizing pre-pulse to 80 mV (P2). Horizontal and vertical scale-bars represent 20 ms and 0.4 nA respectively. iii. The P2/P1 ratio reveals increased facilitation in βarr2-/- but not β-arr2+/+ early post-natal neurons. This difference was enhanced in the presence of GTP-γ-S and absent in neurons lacking both β-arr2 and the μ receptor (μβarr2-/-). B. Adult DRG neurons. i. Similar to the early post-natal DRGs, recordings were made in small DRG neurons from adult mice (highlighted by the arrow, the scale-bar represents 10 μM). ii. Exemplar recordings from adult DRG neurons in which GTP-γ-S was included in the intracellular recording solution, similarly demonstrate an increased current after the depolarizing pre-pulse (P2) in βarr2-/- neurons. Horizontal and vertical scale-bars represent 20 ms and 1.0 nA, respectively. iii. Recordings from adult DRG neurons show a similar effect as early post-natal DRGs in which the P2/P1 ratio was enhanced in β-arr2-/- vs β-arr2+/+ neurons. This effect was enhanced by GTP-γ-S. ***p < 0.0001 vs β-arr2+/+., * p < 0.05 vs β-arr2+/+, #p < 0.05 vs untreated β-arr2-/- recordings.

VDCC inhibition by μ receptors is mediated by Gβγ subunits which bind to VDCCs in a voltage-dependent manner [24]. Inhibition mediated by Gβγ subunits can be reversed by strongly depolarizing pre-pulses leading to facilitation of current mediated by VDCCs [15]. We previously demonstrated that facilitation can be used to monitor the level of inhibitory opioid receptor coupling to VDCCs in the absence of agonist [17]. We used a voltage protocol to generate a current (activated by stepping from -80 to 10 mV) either in the absence (P1) or in the presence (P2) of a depolarizing pre-pulse from -80 to 80 mV (Figure 1A). In our previous study we used small to medium sized neurons [17]. In the current study we measured facilitation in small (classified as having a whole-cell capacitance of < 15 pF) DRG neurons, by determining the P2/P1 ratio (Figure 1A). In agreement with our previous report, the pre-pulse-evoked facilitation was significantly larger (p < 0.005) in β-arr2-/- (1.10 ± 0.02) compared to β-arr2+/+ DRG neurons (1.02 ± 0.01) from early postnatal mice and was augmented when the non-hydrolyzable GTP analog, GTP-γ-S (100 μM) was included in the intracellular recording solution (Figure 1A). An increased facilitation was not seen in DRG neurons from postnatal mice lacking both μ receptors and β-arr2 (1.03 ± 0.02, p < 0.05 Figure 1A), indicating that the μ receptor is required for this phenomenon. Similar to early postnatal DRG neurons, a pre-pulse evoked facilitation was also seen in adult DRG neurons from β-arr2-/- (1.10 ± 0.02) but not β-arr2+/+ (1.03 ± 0.02) mice (p < 0.05, Figure 1B). Again, facilitation was enhanced by the presence of intracellular GTP-γ-S (Figure 1B). Early postnatal DRG neurons were used for all subsequent electrophysiological experiments.

### Characterization of neutral antagonists and inverse agonists in β-arr2-/- DRG neurons

**Figure 2:**
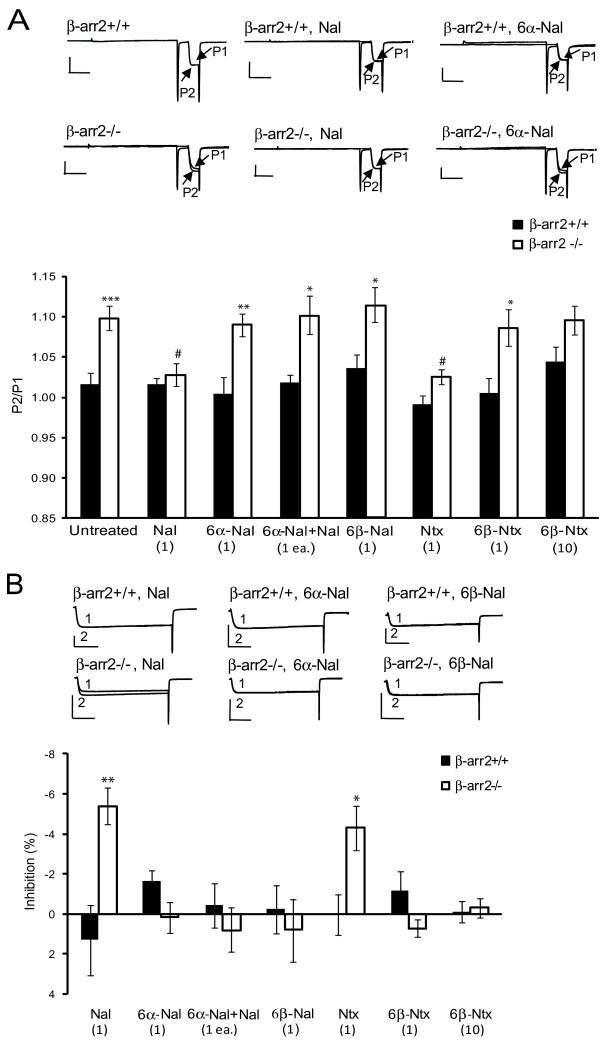
**Constitutive coupling of the μ-receptor with VDCC in β-arr2-/- but not β-arr2+/+ DRG neurons**. A Constitutive coupling of GPCRs to VDCCs can be reversed by inverse agonists but not neutral antagonists. Accordingly the enhanced facilitation ratio in β-arr2-/- vs β-arr2+/+ neurons, shown in Figure 1 and reproduced here for comparison, was inhibited by naloxone (Nal), naltrexone (Ntx), but not by 6α-naloxol, 6β-naloxol (6α-Nal, 6β-Nal), 6β-naltrexol, (6β-Ntx), or by the combination of naloxone with 6α-naloxol (6α-Nal+Nal). These ligands had no effect in β-arr2+/+ neurons. The upper panels show exemplar traces generated by the two-step protocol (scale-bars represent 20 ms (horizontal) and 0.5 nA (vertical) and the data from 12-22 cells are summarized in the underlying graph, with P2/P1 ratio on the ordinate and treatment on the abscissa. ***p < 0.0001 vs β-arr2+/+, *p < 0.05 vs β-arr2+/+, #p < 0.05 vs untreated β-arr2-/-. The concentration of each drug in μM is indicated in parentheses. B. A single-step voltage protocol was used to measure Ba^2+ ^current amplitude in the presence (2) or absence (1) of naloxone, or 6α- and 6β-naloxol, as shown by the exemplar traces in the top panels. Naloxone and naltrexone increased the current amplitude in β-arr2-/- but not β-arr2+/+ neurons, whereas 6α-, 6β-naloxol or 6β-naltrexol, or the combination of naloxone with 6α-naloxol, had no effect on the current amplitude in either cell type. The upper panels show exemplar traces for all conditions. Scale-bars represent 20 ms (horizontal) and 0.5 nA (vertical) and the data for 7-12 cells are summarized in the underlying graph, inhibition (%) shown on the ordinate and treatment on the abscissa. *p < 0.05 vs β-arr2+/+. The concentration of each drug in μM is indicated in parentheses.

We previously demonstrated that facilitation in β-arr2-/- neurons is mediated by constitutively active μ receptors; it is inhibited by the inverse agonist naltrexone, but unaffected by the neutral antagonist CTAP [17]. The relatively poor bioavailability of the peptide CTAP compared to alkaloids [25] prompted us to use voltage-dependent facilitation of VDCC activity in β-arr2-/- neurons as an assay to identify alkaloids that lack negative efficacy for subsequent use *in vivo*. We examined naloxone and naltrexone, previously classified as inverse agonists and 6α-naloxol, 6β-naloxol and 6β-naltrexol classified as neutral antagonists in the reward pathway *in vivo *[11] and in assays of adenylyl cyclase activity *in vitro *[9]. Naloxone (1 μM) reduced facilitation recorded from β-arr2-/- DRG neurons (Figure 2A). By contrast, 6α-naloxol (1 μM), 6β-naloxol (1 μM) and 6β-naltrexol (1 and 10 μM) did not influence facilitation in β-arr2-/- neurons. In agreement with our previous study [17] naltrexone (1 μM), like naloxone, inhibited facilitation. The naloxone-evoked reduction in pre-pulse evoked facilitation in β-arr2-/- neurons was prevented by the co-application of the neutral antagonist, 6α-naloxol (Figure 2A). None of the alkaloids tested affected facilitation in β-arr2+/+ neurons.

### Recovery of VDCC activity in β-arr2-/- neurons by inverse agonists but not neutral antagonists

We also examined the effect of the inverse agonists on currents activated by depolarizing neurons from -80 to 10 mV in the absence of a depolarizing pre-pulse. This approach reveals the amplitude of current recovery by inverse agonist-evoked inhibition of constitutive μ receptor activity. Naloxone (1 μM) and naltrexone (1 μM) caused small enhancements of VDCC mediated currents recorded from β-arr2-/- neurons (5.3 ± 1.0% and 4.3 ± 1.1% of control amplitude) not seen in recordings from β-arr2+/+ neurons (Figure 2B). By contrast, the neutral antagonists 6α-naloxol (1 μM), 6β-naloxol (1 μM) and 6β-naltrexol (1 and 10 μM), had no significant effect on the amplitude of currents recorded from DRG neurons, regardless of genotype. The enhancement of VDCC mediate currents by naloxone (1 μM) was inhibited by co-application of the neutral antagonist, 6α-naloxol (1 μM; Figure 2B).

### Neutral antagonists inhibit agonist-dependent μ receptor coupling to VDCCs

We tested the ability of the neutral antagonists to inhibit agonist induced μ receptor coupling to VDCCs. The specific μ receptor agonist DAMGO (1 μM) inhibited currents recorded from β-arr2+/+ neurons to 28 ± 3% of control current amplitude. By contrast, DAMGO (1 μM) co-applied with the neutral antagonists (1 μM of each) 6α-naloxol, 6β-naloxol, or 6β-naltrexol, barely inhibited currents (2.6 ± 1.0%, 0.1 ± 0.5% and 2.3 ± 1.6%, respectively, n = 4-10). These data confirm that, at the concentrations used, the neutral antagonists bind to μ receptors and inhibit VDCC coupling.

Having determined the relative intrinsic efficacies of naloxone, naltrexone, 6α-naloxol, 6β-naloxol, and 6β-naltrexol *in vitro *we used these bioavailable compounds to investigate the behavioral significance of constitutive μ receptor activity *in vivo*.

### Mice lacking β-arr2 exhibit reduced thermal nociception that is reversed by naloxone and naltrexone

**Figure 3:**
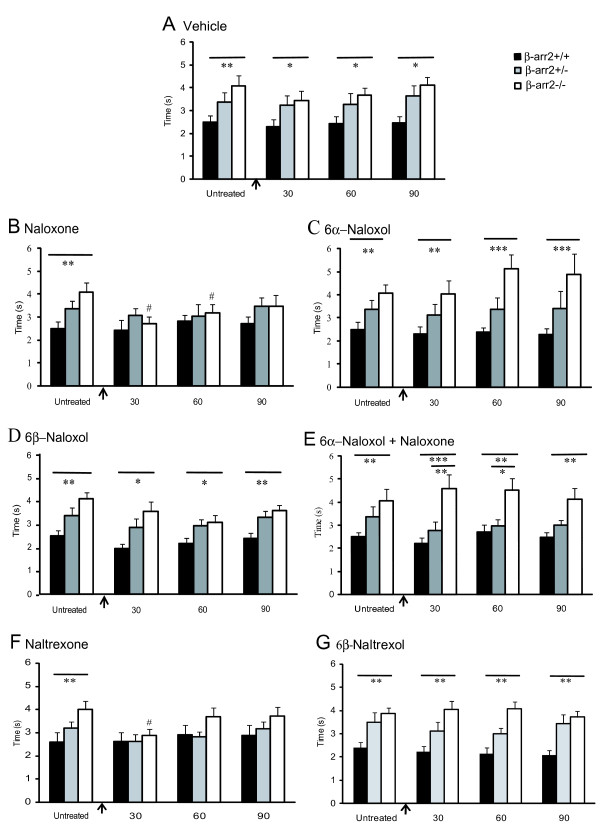
**Increased basal analgesia in β-arr2-/- mice is reversed by μ receptor inverse agonists and unaffected by neutral antagonists**. A. Using time to respond to tail withdrawal from 48°C water, β-arr2-/- mice showed a delayed withdrawal latency compared to β-arr2+/+ mice, (p < 0.05 β-arr2-/- vs β-arr2+/+). B. Naloxone (0.5 mg/kg), reduced the tail withdrawal latency when administered to β-arr2-/- mice 30 and 60 min after injection (#p < 0.05 vs untreated β-arr2-/-), but had no effect in β-arr2+/+ or β-arr2+/- mice. C, D and E. 6α- and 6β-naloxol, (1 mg and 10 mg/kg respectively) and the combination of 6α-naloxol (1 mg/kg) with naloxone (0.5 mg/kg), had no effect on the analgesic profile of the β-arr2-/- mice who continued to show an attenuated response compared with β-arr2+/+ mice (*p < 0.05, **p < 0.005 and ***p < 0.0001 vs β-arr2+/+). F. Similar to naloxone, naltrexone (0.5 mg/kg) reduced the increase in basal analgesia seen in β-arr2-/- mice to wild-type levels 30 min after the injection, but had no effect in β-arr2+/+ or β-arr2+/-mice. **p < 0.001 vs β-arr2+/+ and *p < 0.05 vs β-arr2+/-, #p < 0.05 vs untreated β-arr2-/- mice. G. In contrast, 6β-naltrexol (10 mg/kg) had no effect on the analgesic profile of β-arr2-/-, β-arr2+/-, or β-arr2+/+ mice. **p < 0.001 vs β-arr2+/+ and *p < 0.05 vs β-arr2+/+ mice

Mice lacking β-arr2 have previously been shown to exhibit a delayed thermal response in the tail withdrawal assay [26]. We similarly found an attenuated tail withdrawal response in β-arr2-/- mice that was absent in their β-arr2+/+ littermates (Figure 3A). This enhanced tail withdrawal latency was not seen in mice lacking both μ receptors and β-arr2 (μ-/-/β-arr2-/-: 2.8 ± 0.2 s, μ+/+/β-arr2-/-: 4.6 ± 0.4 s, μ+/+/β-arr2+/+: 2.5 ± 0.3 s, p < 0.05 μ-/-/β-arr2-/- vs μ+/+/β-arr2-/-) indicating that the μ receptor is necessary for the prolonged response to noxious heat.

We examined whether constitutive activity of the μ receptor is responsible for the prolonged withdrawal latency in β-arr2-/- mice. Initially we used the inverse agonist, naloxone (Figure 3B) and the neutral antagonists, 6α-naloxol (Figure 3C) and 6β-naloxol (Figure 3D) using doses of 0.5, 1 and 10 mg/kg for naloxone, 6α- and 6β-naloxol respectively, previously shown to be effective *in vivo *[9,12,13,27,28]. We examined a time course of up to 90 min after each injection, to allow sufficient time for different rates of receptor activation. Naloxone, 30 min after administration, reduced tail withdrawal latency of β-arr2-/- mice from 4.6 ± 0.4s to 3.0 ± 0.3s (Figure 3B), but had no effect in β-arr2+/- or β-arr2+/+ mice (Figure 3B). The mean withdrawal latencies in naloxone treated β-arr2-/- mice at the 30 and 60 min time points were similar to those of β-arr2+/+ mice with or without naloxone administration. Unlike naloxone, neither the neutral antagonist 6α-naloxol nor 6β-naloxol significantly affected tail withdrawal latencies in β-arr2+/+, β-arr2+/- or β-arr2-/- mice (Figure 3C and 3D). However, the co-administration of 6α-naloxol with naloxone abolished the reduction in tail withdrawal latency seen in β-arr2-/- mice treated with naloxone alone, indicating that both drugs have access to μ receptors *in vivo *(Figure 3E). Consistent with these effects being mediated by μ receptors, naloxone had no effect when administered to μ-/-/β-arr2-/- mice 30 min after injection (untreated: 2.81 ± 0.24 s, Nal: 3.03 ± 0.22 s, p = 0.5).

Although naloxone is an inverse agonist at μ receptors, it also binds to δ and κ receptor subtypes [29]. We therefore tested the effect of naltrexone (0.5 mg/kg), an inverse agonist with a similar negative efficacy to that of naloxone [30], but with higher μ receptor selectivity [29] as well as the structurally related neutral antagonist 6β-naltrexol. Since 6β-naltrexol may have a lower affinity for the μ receptor and a slower rate of activation, a higher dose of 6β-naltrexol, effective *in vivo*, was administered [27,31]. We found that similar to naloxone, naltrexone reduced tail withdrawal latency in β-arr2-/- mice, but was without effect in β-arr2+/+ or β-arr2+/- mice 30 min after administration (Figure 3F). By contrast, the neutral antagonist 6β-naltrexol (10 mg/kg) had no effect on tail withdrawal latencies, irrespective of genotype (Figure 3G).

### The β-arr2 gene deletion does not affect all pain modalities

**Figure 4:**
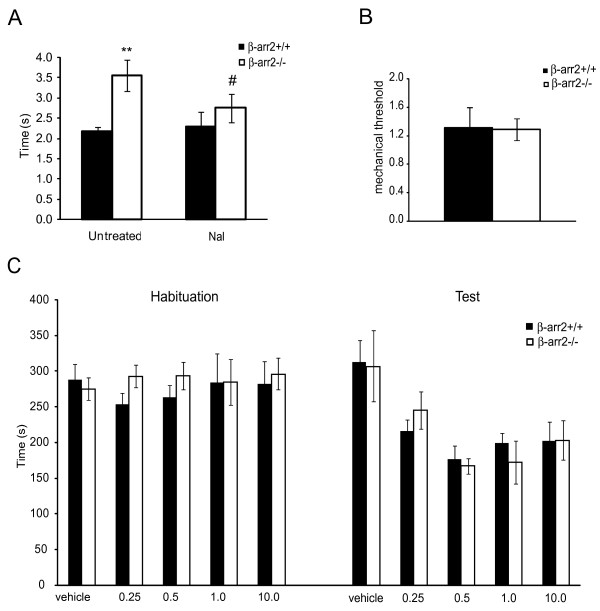
**Constitutive activity of the μ receptor prolongs withdrawal from noxious heat but neither contributes to mechanical pain nor naloxone-mediated conditioned place aversion in β-arr2-/- mice**. A. Similar to the tail-immersion assay, β-arr2-/- mice exhibit a delayed latency to respond in the Hargreaves test of thermal pain. This delay was reversed by naloxone (0.5 mg/kg). **p < 0.001 vs β-arr2+/+, #p < 0.05 vs untreated β-arr2-/- mice. B. However, mechanical pain, as assessed by the response to von Frey filaments, was unaffected by the absence of β-arr2. C Naloxone conditioned place preference is similarly not affected by the absence of β-arr2. After 3 days of conditioning, the aversive effect of naloxone resulted in less time spent in the naloxone-paired chamber for both β-arr2+/+ and β-arr2-/- mice (F_2,34 _= 4.27, p < 0.05). "Habituation" represents the time spent in the naloxone-paired chamber prior to conditioning. "Test" represents the time spent in the naloxone-paired chamber 24 h after the last of the three conditioning sessions. Numbers on the abscissa represent doses (mg/kg) of naloxone administered.

Similar to the tail-immersion response, β-arr2-/- mice also exhibited a delayed response to thermal pain assessed by the Hargreaves test (β-arr2+/+: 2.17 ± 0.11 s, β-arr2-/-: 3.55 ± 0.03 s, p < 0.05; Figure 4A). The response latency was reduced by naloxone (0.5 mg/kg; β-arr2+/+: 2.30 ± 0.34 s, β-arr2-/-: 2.75 ± 0.35 s, p < 0.05 vs untreated β-arr2-/-). However, there was no effect of genotype in the response to mechanical pain assessed by von Frey filaments (β-arr2+/+: 1.31 ± 0.31 g, β-arr2-/-, 1.29 ± 0.15 g; Figure 4B). These data suggest that the enhanced analgesic tone mediated by constitutively active μ receptors in β-arr2-/- mice is specific to the thermal nociceptive pathway.

### Mice lacking β-arr2 exhibit unaltered naloxone conditioned place aversion

In addition to nociception, μ receptors regulate reward and hedonic tone [23,32]. Agonists of the μ receptor induce a conditioned place preference, a measure of reward, while antagonists and inverse agonists produce a conditioned place aversion (CPA), a measure of μ receptor-mediated hedonic tone [12]. As enhanced constitutive activity of the μ receptor induced by morphine treatment leads to increased naloxone aversion [12] we examined whether the enhanced μ receptor constitutive activity seen in β-arr2-/- mice would similarly affect basal hedonic tone. Although naloxone administration (0.25-10 mg/kg sc) produced an aversion to the drug-paired chamber (F_1,61 _= 33.75, p < 0.0001) in a dose-dependent manner (dose × day interaction: F_4,61 _= 6.41, p < 0.001, Figure 4C), there was no effect of genotype. This was evident from the lack of interaction between genotype and day (F_1.,61 _= 0.62, p = 0.44), or between genotype, day and dose (F_4,61 _= 0.15, p = 0.96). There was no overall initial bias between the future drug-paired versus vehicle-paired chamber on habituation day (F_1,61 _= 0.10, p = 0.75), no interaction with dose (F_4,69 _= 0.58, p = 0.68) or genotype (F_1,61 _= 1.29, p = 0.26), and no three-way interaction (F_4,61 _= 0.59, p = 0.67).

## Discussion

Behavioral experiments using inverse agonists and antagonists, characterized in our electrophysiological recordings from DRG neurons, reveal that constitutively active μ receptors in β-arr2-/- mice delay withdrawal responses to noxious heat. By contrast, there was no detectable differences in either the threshold of mechanical pain or the aversive effects of naloxone, in β-arr2+/+ and β-arr2-/- mice. These data suggest that an absence of β-arr2 has pathway specific behavioral effects mediated by constitutively active μ receptors in β-arr2-/- mice.

Our demonstration that constitutively active μ receptors play a role in thermal nociception in β-arr2-/- mice provides a mechanism for the phenomenon of prolonged basal tail withdrawal latencies observed in previous studies using these animals [26]. It is well established that μ opioid receptors are located on primary afferent neurons that transmit pain evoked by noxious heat [32]; therefore it is perhaps not surprising that enhanced constitutive μ receptor activity affects thermal nociception. It is interesting that, unlike the antinociceptive effect of morphine, which succumbs to tolerance [18,32], analgesia mediated by constitutively active μ receptors appears to persist providing a long lasting basal analgesic tone in β-arr2-/- mice.

It is likely that the lack of effect of constitutive μ receptor activity on paw withdrawal using the von Frey assay in β-arr2-/- mice can be explained by an absence of μ receptors in mechanical nociceptive neurons which appear to predominantly express delta receptors [33]. However, pathway specific effects of constitutive μ receptor activity may also be explained by a disparate influence of β-arr2 in different neuronal populations. The ability of morphine to induce μ receptor internalization in striatal neurons, but not spinal neurons, for example, has been attributed to differing properties of β-arr2 in these cell types [34].

A previous study also observed differences in basal nociception in β-arr2-/- mice using different behavioral assays [26]. In keeping with our findings, tail withdrawal from immersion in hot water was prolonged in β-arr2-/- compared to β-arr2+/+ mice. However, there was no difference in paw responses to heat generated using a hotplate. The latter assay quantifies the response to heat by scoring paw lifting and licking and the basal levels of these behaviors appear somewhat variable between studies [18,26]. The different anatomical locations of the pain does not account for differences between tail and paw responses since we observed prolonged basal paw withdrawal in β-arr2-/- compared to β-arr2+/+ mice using the Hargreaves assay. However, it is likely that the more complex pain behavior generated by the hot-plate assay involves a greater input from the CNS perhaps recruiting neuronal pathways that are unaffected by constitutive μ receptor activity. This could account for a lack of effect of genotype on basal responses to the hotplate.

The possibility that constitutive μ receptors in β-arr2-/- mice play a greater role in peripherally-or spinally-mediated responses compared to centrally-mediated responses is supported by our findings using the conditioned place aversion assay of basal hedonic tone. Stimulation of hedonic tone by prolonged exposure to morphine causes an enhanced aversive response to the inverse agonist naloxone but not to the neutral competitive antagonists [11]. By contrast, there was no difference between the aversive responses to naloxone of β-arr2-/- and β-arr2+/+ mice. These data suggest that the nature of the regulation μ receptors by β-arr2 within the reward pathway differs from that of the peripheral pain pathway. In keeping with this idea β-arr2-/- mice exhibit delayed analgesic tolerance to chronic morphine without altered centrally-mediated morphine dependence [35].

Our study reveals that there are key differences between ligand-independent constitutive signaling and agonist-induced μ receptor activity. The former causes a low level basal inhibition of VDCC activity in DRG neurons and this is associated with a modest but sustained antinociception in β-arr2-/- mice. By contrast, the latter produces a more profound VDCC inhibition [17] resulting in strong analgesia, the duration of which is limited by the adaptive process of tolerance and the opponent process of hyperalgesia [14,36]. Furthermore, unlike μ receptor activation by morphine, which can lead to reward and dependence, constitutive μ receptor activity, induced by the absence of β-arr2, appears not to disrupt hedonic tone.

We previously demonstrated that inhibition of Src in β-arr2+/+ neurons mimics the enhancement of μ receptor constitutive activity seen in β-arr2-/- neurons [17]. β-arr2, Src and Akt form a signaling complex in vivo that is disrupted in β-arr2-/- mice [37]. μ Receptor activation stimulates Akt [38] a process implicated in cross talk with the NMDA receptor and tolerance [39,40]. Further studies are required to examine whether targeting the signaling pathway mediated by the β-arr2 complex can produce sustained analgesia through constitutive μ receptor activity.

## Competing interests

The authors declare that they have no competing interests.

## Authors' contributions

This study was designed by TGH, CJE, NTM and WW. HL, MM and AP carried out the behavioural experiments. WW performed the electrophysiology experiments, analyzed all data and drafted the manuscript. CJE, NTM and TGH obtained funding and revised the manuscript, the final version of which was read and approved by all authors.
